# Effect of Genital Herpes on Cervicovaginal HIV Shedding in Women Co-Infected with HIV AND HSV-2 in Tanzania

**DOI:** 10.1371/journal.pone.0059037

**Published:** 2013-03-13

**Authors:** Jim Todd, Gabriele Riedner, Leonard Maboko, Michael Hoelscher, Helen A. Weiss, Eligius Lyamuya, David Mabey, Mary Rusizoka, Laurent Belec, Richard Hayes

**Affiliations:** 1 Department of Population Health, London School of Hygiene & Tropical Medicine, London, United Kingdom; 2 National Institute for Medical Research - Mbeya Medical Research Programme, Mbeya, Tanzania; 3 Department of Infectious Diseases and Tropical Medicine, Klinikum, Ludwig-Maximilians-University, Munich, Germany; 4 Muhimbili University College of Health Sciences, Dar es Salaam, Tanzania; 5 Laboratoire de Microbiologie, hôpital Européen Georges Pompidou, Paris, France; 6 Faculté de Médecine Paris Descartes, Université Paris Descartes (Paris V), Paris, France; University of Toronto, Canada

## Abstract

**Objectives:**

To compare the presence and quantity of cervicovaginal HIV among HIV seropositive women with clinical herpes, subclinical HSV-2 infection and without HSV-2 infection respectively; to evaluate the association between cervicovaginal HIV and HSV shedding; and identify factors associated with quantity of cervicovaginal HIV.

**Design:**

Four groups of HIV seropositive adult female barworkers were identified and examined at three-monthly intervals between October 2000 and March 2003 in Mbeya, Tanzania: (1) 57 women at 70 clinic visits with clinical genital herpes; (2) 39 of the same women at 46 clinic visits when asymptomatic; (3) 55 HSV-2 seropositive women at 60 clinic visits who were never observed with herpetic lesions; (4) 18 HSV-2 seronegative women at 45 clinic visits. Associations of genital HIV shedding with HIV plasma viral load (PVL), herpetic lesions, HSV shedding and other factors were examined.

**Results:**

Prevalence of detectable genital HIV RNA varied from 73% in HSV-2 seronegative women to 94% in women with herpetic lesions (geometric means 1634 vs 3339 copies/ml, p = 0.03). In paired specimens from HSV-2 positive women, genital HIV viral shedding was similar during symptomatic and asymptomatic visits. On multivariate regression, genital HIV RNA (log_10_ copies/mL) was closely associated with HIV PVL (*β* = 0.51 per log_10_ copies/ml increase, 95%CI:0.41–0.60, p<0.001) and HSV shedding (*β* = 0.24 per log_10_ copies/ml increase, 95% CI:0.16–0.32, p<0.001) but not the presence of herpetic lesions (*β* = −0.10, 95%CI:−0.28–0.08, p = 0.27).

**Conclusions:**

HIV PVL and HSV shedding were more important determinants of genital HIV than the presence of herpetic lesions. These data support a role of HSV-2 infection in enhancing HIV transmissibility.

## Introduction

*Herpes simplex* virus type-2 (HSV-2) infection is lifelong and a common cause of genital ulcers [1]–[3]. There is strong epidemiological evidence that HSV-2 infection increases the risk of HIV acquisition [4]–[6]. The potential effect of HSV-2 on HIV infectivity is also of interest. *In vitro* studies provide evidence of the effect of HSV-2 infection on HIV replication [7]–[9] and epidemiological studies link acute episodes of genital herpes to temporal increases in HIV plasma viral load [10]. A prospective study in Ugandan discordant couples has shown that the risk of HIV transmission is increased in the presence of genital ulcer disease when the plasma viral load exceeds 1,700 copies/mL [11]. Observational studies have shown an association between HSV-2 genital shedding and HIV genital shedding in some cases but not others [12]–[15]. Trials of HSV suppressive therapy in HIV positive women have shown varying impact on cervicovaginal HIV shedding [16]–[22] while a trial of suppressive therapy delivered to the HIV positive partner in discordant couples found no effect on HIV transmission to their HIV negative partners [23].

The frequency of recurrent episodes of clinical herpes and of subclinical viral shedding varies both between individuals and over time within the same individual [24], [25]. While research supports an association between HSV infection and HIV shedding, it is less clear whether any effect on HIV infectivity is restricted primarily to clinical episodes of genital herpes or if HIV transmission is also enhanced during asymptomatic HSV-2 infection [26]. This is particularly important since asymptomatic HSV shedding is observed to occur on up to 28% of days in infected individuals [27].

The objectives of this study were to compare the presence and quantity of cervicovaginal HIV in HIV seropositive women living in Mbeya Region, Tanzania with clinical episodes of herpes, subclinical HSV-2 infection and without HSV-2 respectively; to examine the association between genital shedding of HIV and HSV-2; and to investigate factors influencing the quantity of HIV genital shedding.

## Methods

### Subjects and Study Methods

As part of a study of the determinants of HIV super-infection in Mbeya Region, Tanzania, an open cohort of 600 female barworkers aged 18 to 35 years was established in late 2000. Study procedures have been described previously [28]. Briefly, behavioural and biological data were collected at baseline and during 3-monthly follow-up visits for up to 30 months. In this paper we present data collected from HIV infected participants between October 2000 and March 2003.

### Laboratory Methods (for All Women)

#### Serological testing and plasma viral load

A diagnostic algorithm including two different enzyme-linked immunosorbent assays (HIV-Determine, Abott Laboratories, USA and Enzygnost HIV 1+2 plus, Behring, Germany) and a Western Blot assay (HIV Blot 2.2, Genelabs Diagnostics, Singapore) was used to determine HIV serostatus. Type-specific serological testing for HSV-2 was also performed by enzyme immune assay (Kalon Biological Ltd, Aldershot, UK). Syphilis testing was carried out using the Serodia *Treponema pallidum* particle agglutination assay (TPPA, Fujirebio Inc, Tokyo, Japan) and the rapid plasma reagin test (RPR, VD25, Murex Diagnostics Ltd, Cambridge, UK).

HIV-1 plasma viral load (PVL) was quantified by transcription polymerase chain reaction (PCR) (Versant 3.0 assay, Bayer diagnostics, Emeryville, California), with a detection threshold of 50 HIV-RNA copies/ml.

#### Genital ulcer specimens

A single dry swab was taken from genital ulcers and multiplex PCR (M-PCR) was used for the detection of *T pallidum*, *H ducreyi* and HSV [29]. Herpetic lesions were defined as ulcers or vesicles confirmed by M-PCR as positive for HSV.

#### Cervicovaginal specimens and vaginal swabs

Cervicovaginal secretions (CVS) were collected from all women at every study visit using standard vaginal lavage procedures [30], by washing with 5 ml of phosphate-buffered saline. After centrifugation, the cell-free supernatant and cell pellet were stored separately at −80°C until use. Real time PCR was used to quantify HIV-1 RNA in the acellular fraction and HIV-1 DNA in the cell pellet of genital secretions as previously described [31] using primers and probes synthesised by Eurogentec (Eurogentec SA, Seraing, Belgium). HSV DNA (Roche Molecular Diagnostics) was also quantified in the acellular fraction of secretions by real time PCR [32]. Detection thresholds were 250 copies/ml for HIV-1 RNA and HSV DNA, and 5 copies/10^6^ cells for HIV-1 DNA. Cervicovaginal specimens were also tested for prostate specific antigen (PSA) using the VITROS immunodiagnostic product PSA reagent and calibrator packs on the automaton Ortho-Clinical Diagnostics VITROS Eci (Ortho-Clinical diagnostics, Issy Les Moulineaux, France), and for traces of haemoglobin using Multistix 8 SG strips (Bayer Diagnostics, Puteaux, France).

Vaginal swabs were tested for *Trichomonas vaginalis* by direct microscopy of a wet mount preparation and *Candida albicans* by wet mount preparation and gram stain.

### Study Design for Shedding Sub-study

CVS specimens collected from HIV seropositive women at 3-monthly study visits were excluded from analyses if (i) the woman had a genital lesion of non-herpetic origin (12 specimens were excluded for confirmed syphilis and 7 specimens excluded for chancroid), (ii) there were traces of haemoglobin in the specimen or (iii) the specimen was positive for PSA. Specimens from (i) were excluded as HSV shedding may have been influenced by other ulcerative STIs, and those from (ii) and (iii) were excluded as any HIV-RNA or HIV-DNA found in the genital tract may not have been attributable to genital shedding from the woman. Specimens were categorised into four study groups according to the woman’s HSV-2 serostatus and the presence or absence of confirmed herpetic genital lesions at the time of specimen collection. Women who experienced PCR-confirmed herpetic lesions at one or more study visits contributed to two study groups: Group 1 (“clinical herpes”) included CVS specimens collected in the presence of herpetic lesions; Group 2 (“subclinical herpes”), included specimens taken from the same women at follow-up examinations at least 6 months after or 6 months before the visits at which herpetic lesions were detected. In Group 3 (“never lesions”) a random sample of specimens were taken from HSV-2 seropositive women who never had herpetic genital lesions or a history of genital lesions at any of the study visits. Group 4 (“HSV-2 negative”) was randomly chosen from specimens taken from women not infected with HSV-2.

### Statistical Analyses

Differences between study groups in proportions of CVS specimens with HIV-1 (RNA and DNA) and HSV DNA above the detection threshold were tested using random effects logistic regression modelling to account for correlations within subjects. Analyses of quantity of genital viral shedding were restricted to specimens with viral loads above the threshold of detection. Logarithmic (log_10_) transformations were applied to plasma and genital viral loads for HIV and HSV-2 to obtain approximately normal distributions, geometric means (GM), and 95% confidence intervals (95%CI). Differences in mean viral loads between groups were assessed using a random effects linear regression model to account for correlations within subjects. Correlations between viral loads (plasma and genital) were assessed using Spearman’s rank correlation coefficient.

The effect of genital herpetic lesions on HIV and HSV shedding was assessed by carrying out matched pair chi-squared tests on detection of virus, and a paired t-test on the quantity of shedding, for paired samples collected from the same women during clinical and subclinical phases of herpes, randomly selecting a single sample result if more than one eligible sample was available. Sample size calculations used the log scale for HIV shedding, assuming a within-women difference of 0.3 log copies/mL between episodes with herpetic lesions and those without herpetic lesions, and a standard deviation of 0.6 log copies/mL. With 60 women providing paired samples, the analysis had a 80% power to detect a difference of 0.3 as significant at the 5% level (which corresponds to a doubling of the HIV shedding in women with clinical lesions).

Initial analyses suggested that HSV shedding was a strong determinant of genital HIV shedding. We regrouped specimens of HSV-2 seropositive women in the subclinical and never lesions groups according to the presence of HSV shedding (asymptomatic HSV shedders) or absence of HSV shedding (HSV non-shedders). We then used random effects linear regression, accounting for multiple observations from the same individual, to examine the association of log-transformed HIV genital viral loads with HSV shedding status as well as other potential risk factors, including age, laboratory-diagnosed non-ulcerative sexually transmitted infections (STI), signs or symptoms of sexually transmitted diseases (STD), enlarged lymph nodes and HIV plasma viral load. In this analysis, specimens with HIV or HSV genital viral load below the threshold of detection were assigned a value of half the threshold.

Analyses were performed using Stata 11.0 (Stata Corporation, Texas, USA). The study was approved by the national ethics review committee of the National Institute for Medical Research of the United Republic of Tanzania and the LSHTM Ethics Committee (Approval 668). It was conducted within the framework of the Mbeya Medical Research Programme. All participants provided written, informed consent to participate in the study.

## Results

### Characteristics of Participants

Analyses were based on 221 eligible CVS samples from 131 HIV seropositive women. The four study groups comprised 57 women at 70 study visits with clinically manifest genital herpes (Group 1), 39 of the same women at 46 study visits when they did not have visible ulcers (Group 2), 55 HSV-2 seropositive women at 60 study visits who were never observed with herpetic lesions (Group 3), and 18 women at 45 study visits who were HSV-2 seronegative (Group 4) (Table 1).

**Table 1:** Characteristics of participants and cervicovaginal viral shedding by study group.

Factor	Study group				
	Clinical herpes	Subclinical herpes	Never lesions	HSV-2 negative	
**Women**	**N = 57**	**N = 39**	**N = 55**	**N = 18**	**p-value** 1
Age Mean (SD)	28.1 (4.20)	28.8 (4.13)	27.2 (4.27)	24.6 (4.34)	0.001 2
**Hormonal contraceptive use**	17 (30%)	8 (21%)	20 (36%)	7 (39%)	0.35
**Specimens**	N = 70	N = 46	N = 60	N = 45	
**Plasma HIV-1 RNA**					
Geo mean (95% CI)	24400 (16400–36300)	16300 (9900–26900)	10550 (6900–16100)	5830 (3540–9600)	<0.001^2^
**Presence of STI (%)**					
TV	9 (13%)	4 (9%)	8 (13%)	2 (4%)	0.49
CA	21 (30%)	9 (20%)	27 (45%)	16 (36%)	0.08
TPPA	32 (46%)	22 (48%)	33 (55%)	8 (18%)	<0.001
RPR	11 (16%)	5 (11%)	9 (15%)	2 (4%)	0.28
Warts	10 (14%)	9 (20%)	5 (8%)	3 (7%)	0.8
Cervicitis	9 (13%)	7 (15%)	9 (15%)	4 (9%)	0.88
Vaginal discharge	28 (40%)	21 (46%)	27 (45%)	28 (62%)	0.2
Lymphadenopathy	19 (27%)	8 (17%)	8 (13%)	6 (13%)	0.19
**CVS HIV RNA**	66 (94%)	41 (89%)	49 (82%)	33 (73%)	0.13
Geo mean^3^ (95% CI)	3339 (2410–4626)	2460 (1653–3664)	1908 (1319–2758)	1634 (1096–2436)	0.025 2
**CVS HIV DNA** 4	63 (100%)	43 (98%)	43 (90%)	43 (100%)	0.002
Geo mean^3^ (95% CI)	252 (182–349)	152 (115–201)	141 (93–213)	143 (101–203)	0.025^2^
**CVS HSV DNA**	54 (77%)	9 (20%)	7 (12%)	–	<0.001
Geo mean^3^ (95% CI)	4260 (2384–7613)	4336 (1245–15102)	1392 (288–6729)	–	0.37^2^

SD, standard deviation; STI, sexually transmitted infection; TV, *Trichomomas vaginalis*; CA, *Candida albicans*; TPPA, *Treponema pallidum* particle agglutination assay; RPR, rapid plasma reagin test; Geo mean, geometric mean in copies/ml (HIV RNA and HSV DNA) or copies/10^6^ cells (HIV DNA); CVS, cervico-vaginal lavage specimen.

1 p-value from random effect logistic regression accounting for correlation within subjects.

2 p-value from random effects linear model on log-transformed viral load accounting for correlation within subjects.

3 Excluding data below the minimum detectable threshold.

4 Missing values for 7 with clinical herpes, 2 with sub-clinical herpes, 12 with never lesions, and 2 HSV-2 negative.

Thirty-five women contributed to both Groups 1 and 2; four other women in Group 2 were not sampled at the time of clinical herpes. Three women were HSV-2 seronegative (Group 4) and later seroconverted, two with an episode of clinical herpes (Group 1) and one with no episode of clinical herpes (Group 3). Thirty-three women contributed more than one specimen to the same study group (up to 7 specimens from one HSV-2 seronegative woman).

Mean age was around 28 years for HSV-2 positive women and 25 years for HSV-2 negative women. Slightly higher proportions of women were using hormonal contraception among those who had never had lesions, and those who were HSV-2 negative, but this difference was not statistically significant (p = 0.35).

Geometric mean PVL was lowest (5,830 copies/ml) in samples from women uninfected with HSV-2 and highest (24,400 copies/ml) in samples from women with confirmed herpetic lesions. TPPA seropositivity, indicating past or current syphilis, was also lower in the HSV-2 uninfected group (p<0.001), and *C Albicans* was most common in the never lesions group (p = 0.08), but other STI or clinical signs of STD at the time of specimen collection did not differ significantly by study group (Table 1).

### Genital Shedding of HIV and HSV by Study Group

The prevalence of detectable genital HIV RNA was lowest in the HSV-2 negative group (73%) and highest in the clinical herpes group (94%) (Table 1). The genital HIV RNA viral load in those with detectable virus showed a similar trend across the four groups, with the lowest quantity in the HSV-2 negative group (GM = 1634 copies per ml) and the highest in the clinical herpes group (GM = 3339 copies per ml) (p<0.025).

The prevalence of detectable genital HIV DNA also varied across study groups (p = 0.002). The pattern was less clear than for HIV RNA, but the highest viral load was seen in the clinical herpes group (GM = 252 copies per 10^6^ cells) and the lowest in the never lesions group (GM = 141 copies per 10^6^ cells) (Table 1).

All women with detectable genital HIV RNA also had detectable plasma HIV RNA. Among women with detectable genital HIV RNA, quantities of plasma and genital HIV RNA were significantly correlated (N = 189, Spearmańs r = 0.49, p<0.001).

As expected, the prevalence of detectable genital HSV DNA varied substantially between the three study groups of HSV-2 positive women, with a much higher prevalence among women with clinical herpes (77%) than in the subclinical herpes (20%) and never lesions (12%) groups (p<0.001). HSV DNA viral load showed a similar, though non-significant trend (Table 1).

Of the 35 women who had specimens taken during clinical episodes of herpes and also at visits when they were asymptomatic, 30 (86%) had detectable HIV RNA on both occasions, while all 29 women with paired specimens analysed for HIV DNA were positive on both occasions. There was no evidence of a difference in the quantity of genital HIV shed between clinical and subclinical phases of herpes (Table 2). Although 23 (66%) of the 35 women had detectable genital HSV DNA during clinical herpes, only 7 (20%) had detectable HSV DNA during the subclinical phase (p<0.001), with 5 (14%) shedding on both occasions.

**Table 2:** Cervicovaginal HIV-1 and HSV viral load in 35 women during the clinical and subclinical stages of herpes.

Viral shedding	N	Number (%) with detectable virus Geometric mean (95% CI)		p-value^1^	p-value^2^
		Clinical herpes	Subclinical herpes		
**CVS HIV-1 RNA** 3 **Number (%)**	35	31 (89%)	32 (91%)	0.56	
**CVS HIV-1 RNA** 3 **Geometric mean (95% CI)**	35	3137 (1914–5143)	2800 (1804–4345)		0.72
**CVS HIV-1 DNA** 3,4 **Number (%)**	29	29 (100%)	29 (100%)	1.0	
**CVS HIV-1 DNA** 3,4 **Geometric mean (95% CI)**	29	157 (90–221)	157 (110–221)		0.99
**CVS HSV DNA** 3 **Number (%)**	35	23 (66%)	7 (20%)	0.0003	
**CVS HSV DNA** 3 **Geometric mean (95% CI)**	35	3369 (1210–9380)	4236 (1351–13278)		0.24

Analyses restricted to women with samples in both the clinical and the subclinical groups.

1 p-value from matched pairs chi-squared test.

2 p-value from matched pairs t-test on logged values.

3 Geometric mean in copies/ml (HIV RNA and HSV DNA) or copies/10^6^ cells (HIV DNA), excluding data below the minimum detectable threshold at half the detectable level.

4 Six women did not have HIV DNA samples tested (five with clinical herpes, and one with subclinical herpes) and are excluding from this comparison.

### Association between Genital HIV Shedding and Genital HSV Shedding

To explore whether signs of clinical herpes or HSV-2 viral shedding were more important determinants of HIV shedding, we re-categorised the specimens into four new groups: clinical herpes, HSV-2 seropositive with detectable genital HSV DNA shedding (asymptomatic HSV shedders), HSV-2 seropositive without detectable genital HSV shedding (HSV non-shedders at that visit), and HSV-2 seronegative. In all four groups, the majority of specimens had detectable HIV RNA and HIV DNA, and the prevalence was highest in samples taken during clinical or subclinical shedding episodes (Table 3). Overall, the prevalence and quantity of genital HIV-1 RNA and DNA shedding differed by sub-group (Table 3). Genital HIV-1 RNA and DNA viral loads were at least as high among asymptomatic HSV shedders as among specimens collected during episodes of clinical herpes. Similarly genital viral load was similar between HSV non-shedders and HSV-2 seronegative women.

**Table 3:** Cervicovaginal HIV-1 RNA, HIV-1 DNA and HSV DNA viral load by HSV-2 group.

	HSV-2 group						
Genital viralShedding	ClinicalHerpes		SubclinicalHSV shedders	SubclinicalNon shedders	HSV-2 negative	p-value^1^	
**HIV-1 RNA**	**N = 70**		**N = 16**	**N = 90**	**N = 45**		
Number (%)	66 (94%)		16 (100%)	74 (82%)	33 (73%)	0.11	
Geo mean 2 (95% CI)	3339 (2410–4626)		4326 (2076–9012)	1840 (1392–2433)	1634 (1096–2436)	0.002	
**HIV-1 DNA**	**N = 63**		**N = 15**	**N = 77**	**N = 43**		
Number (%)	63 (100%)	15 (100%)		71 (90%)	43 (100%)		0.04
Geo mean 2 (95% CI)	252 (182–349)	260 (131–517)		130 (100–203)	143 (101–203)		0.003
**HSV DNA**	**N = 70**	**N = 16**					
Number (%)	54 (77%)	16 (100%)		NA	NA		0.03
Geo mean 2 (95% CI)	4260 (2383–7613)	2637 (1064–6541)		NA	NA		0.4

IQR, interquartile range; CI, confidence interval.

1 p-values from random effects logistic regression model, or random effects linear regression model.

2 Geometric mean in copies/ml (HIV RNA and HSV DNA) or copies/10^6^ cells (HIV DNA), excluding data below the minimum detectable threshold level.

**Figure 1:**
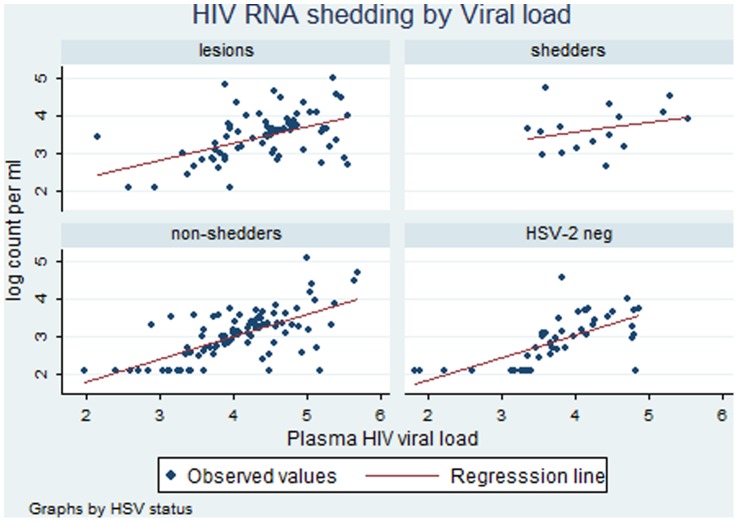
Relationship between HIV RNA genital shedding and HIV plasma viral load in women with herpetic lesions, asymptomatic women with and without HSV genital shedding and HSV-2 seronegative women.

**Figure 2:**
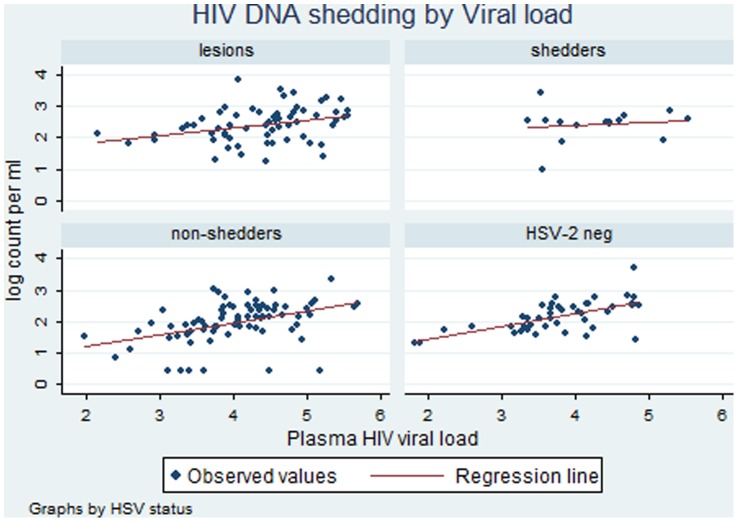
Relationship between HIV DNA genital shedding and HIV plasma viral load in women with herpetic lesions, asymptomatic women with and without HSV genital shedding and HSV-2 seronegative women.

Among specimens with HSV DNA shedding (with or without lesions), the genital viral loads of HIV RNA and HSV DNA were positively correlated (n = 86, r = 0.47, p<0.001). The correlation of genital HIV DNA and HSV DNA was weaker (n = 78, r = 0.24, p = 0.03), as was the correlation between plasma HIV RNA and genital HSV DNA (n = 78, r = 0.24, p = 0.03). A positive correlation between genital and plasma viral loads of HIV was seen in all women (Fig. 1 and Fig. 2). While the slopes of the regression lines were similar, the intercept was higher in the clinical herpes and HSV shedding groups indicating that, for any given PVL, the genital shedding of HIV was higher in those who were also shedding HSV-2.

### Risk Factors for Genital HIV Shedding

Genital HIV RNA viral load increased with quantity of HIV PVL (p<0.001) and presence and quantity of genital HSV DNA (Table 4). Although the quantity of genital HIV RNA was higher in specimens from HSV-2 seropositive women (p = 0.009) and those with herpetic lesions (p = 0.002), the association with herpetic lesions was no longer statistically significant after adjusting for age, PVL and the quantity of genital HSV DNA (Table 4). The quantity of genital HIV RNA was unrelated to the presence of STI, signs or symptoms of other STD or enlarged lymph nodes (data not shown), or to the use of hormonal contraception.

**Table 4:** Association between cervicovaginal HIV-1 RNA and HIV-1 DNA viral load and potential risk factors.

		Age adjusted		Adjusted for age and other factors^1^	
Factors		Coefficient (95% CI)	p-value	Coefficient (95% CI)	p-value
**Genital HIV-RNA shedding**	**No with characteristic**	N = 221		N = 221	
**Age group** (years)			0.61		0.6
≤24	50	0		0	
25–29	101	+0.04 (−0.21, 0.30)		−0.06 (−0.24, 0.11)	
≥30	70	+0.03 (−0.25, 0.31)		−0.12 (−0.30, −0.07)	
**Hormonal contraceptive use**	84	−0.07 (−0.29, 0.14)	0.5	+0.05 (−0.10, 0.19)	0.54
**Plasma HIV-1 RNA**	All	+0.57 (0.48, 0.67)	<0.001	+0.51 (0.41, 0.60)	<0.001
**HSV-2 seropositive**	176	+0.37 (0.09, 0.65)	0.009	+0.65 (0.40, 0.91)	<0.001
**Presence of herpetic lesions**	70	+0.31 (0.12, 0.50)	0.002	−0.10 (−0.28, 0.08)	0.27
**Genital HSV DNA shedding**					
Any HSV DNA shedding 2	70	+0.53 (0.34, 0.71)	<0.001	+0.04 (−0.22, 0.29)	0.77
Quantitative 3	All	+0.13 (0.05, 0.22)	0.002	+0.24 (0.16, 0.32)	<0.001
**Genital HIV-DNA shedding**	**No with characteristic**	N = 198		N = 198	
**Age group** (years)			0.61		0.5
≤24	50	0		0	
25–29	101	+0.21 (−0.03, 0.46)		+0.20 (−0.00, 0.40)	
≥30	70	+0.13 (−0.14, 0.40)		+0.06 (−0.16, 0.27)	
**Hormonal contraceptive use**	84	−0.03 (−0.24, 0.18)	0.8	−0.01 (−0.16, 0.18)	0.9
**Plasma HIV-1 RNA**	All	+0.35 (0.25, 0.46)	<0.001	+0.33 (0.23, 0.44)	<0.001
**HSV-2 seropositive**	176	−0.09 (−0.37, 0.19)	0.5	+0.14 (−0.14, 0.43)	0.33
**Presence of herpetic lesions**	70	+0.26 (0.08, 0.44)	0.004	+0.11 (−0.09, 0.31)	0.29
**Genital HSVDNA shedding**					
Any HSV DNA shedding 2	70	+0.31 (0.13, 0.49)	<0.001	+0.12 (−0.17, 0.40)	0.42
Quantitative 3	All	+0.14 (0.06, 0.22)	<0.001	+0.15 (+0.06, 0.24)	<0.001

Linear coefficient on log_10_ scale; p-value from Wald test from a generalised least square random effects model.

1 Adjusted for age, plasma viral load, quantitative cervico-vaginal shedding of HSV and HSV-2 seropositivity.

2 Binary variable comparing samples from women with HSV DNA genital shedding and samples from women with no shedding.

3 Quantitative analysis: Assessing the effect of a unit log increase in HSV DNA genital shedding. Women who were HSV-2 seronegative and women who were HSV-2 seropositive with undetectable HSV genital shedding were given a value of half the detectable threshold.

Similarly, genital HIV DNA viral load increased with both HIV PVL (p<0.001) and genital HSV DNA viral load (Table 4). Although genital HIV DNA was more commonly detected in the presence of clinical herpes (p = 0.004), this association was no longer statistically after adjusting for age, PVL and the quantity of genital HSV DNA. The quantity of genital HIV DNA was unrelated to the presence of STI, signs or symptoms of other STD or enlarged lymph nodes (data not shown), or to the use of hormonal contraception.

## Discussion

In this study we determined the frequency and quantity of cervicovaginal HIV and HSV using specimens collected from 131 HIV infected women who were followed up every three months for up to 30 months. The prevalence of detectable genital HSV DNA in asymptomatic HIV positive women was similar to studies from elsewhere in Africa [33] and, as in other studies, there was a strong correlation between genital and plasma HIV viral loads [15].

The main objective of our study was to determine whether genital shedding of HIV occurred more frequently in the presence of genital herpetic lesions. A strength of the study was the long follow-up period, providing an opportunity to observe women both during and between clinical episodes of herpes. Clinical herpes was associated with higher levels of genital HIV shedding, but only partially as there were episodes of herpetic ulceration where HSV genital shedding was not recorded, and conversely episodes of HSV genital shedding in asymptomatic women. In the multivariate analysis, we clearly saw that herpetic ulcers were not independently associated with genital shedding of HIV RNA (coefficient = −0.10, p = 0.27), whereas there remained a clear association between HSV genital shedding and genital shedding of HIV RNA (coefficient = +0.24, p<0.001), suggesting that this increase was related to the quantity of genital HSV and the HIV plasma viral load rather than to the presence or absence of herpetic lesions. In paired specimens taken from women with clinical herpes and from other study visits when they were asymptomatic, no difference was seen in HIV genital shedding at the two visits. When the original study groups were rearranged to examine the effect of genital HSV shedding, there was a clear difference in HIV genital shedding between HSV shedders and non-shedders, but no additional increase in HIV shedding in those with clinical herpes. Similarly, when risk factors for HIV genital shedding were examined in a multivariate regression analysis, the quantity of genital HIV RNA or DNA was significantly associated with HIV plasma viral load and HSV shedding, but after adjustment for these factors was not associated with clinical herpes.

Our findings are consistent with results from a study carried out in Bangui, Central African Republic, where among HIV positive women shedding HSV, there was a significant correlation between genital HIV RNA shedding and genital shedding of HSV [14]. A similar positive correlation between cervical HSV shedding and the quantities of cervical HIV-1 RNA and DNA was seen in a cross-sectional study of 200 women attending family-planning clinics in Mombasa, Kenya, and this was significant even after controlling for differences in CD4 count and plasma viral load [13]. Baseline data from a randomised controlled trial of herpes episodic treatment in Ghana and Central African Republic showed that, among 180 HSV-2/HIV-1 co-infected women, genital HIV RNA was detected more frequently and at higher median viral loads among women with genital HSV-2 infection (i.e. HSV-2 ulcers or HSV-2 in CVS) than women without any genital HSV-2 infection [34]. However, in a study of 214 Zimbabwean sex workers, genital HIV-1 RNA shedding increased with HIV plasma viral load but did not differ in women with or without HSV shedding, although the HSV shedding was not quantified [15]. A study in men and women in Thailand showed no association between the quantities of HIV shed and the presence of either HSV shedding or herpetic lesions [35].

The observational design of these studies limits inferences about causality, but stronger evidence concerning the effect of herpes on HIV infectivity comes from seven recently completed randomised controlled trials of herpes suppressive therapy. Four trials, using valacyclovir or high dose acyclovir (800 mg b.i.d.), found significant reductions in PVL as well as significant reductions in rectal and cervicovaginal HIV-1 RNA concentrations [16]–[19]. Trials of acyclovir 400 mg b.i.d have shown less effect. Two found no reduction in cervico-vaginal HIV-1 RNA detection [20], [21], possibly attributable to sub-optimal adherence and the other found a reduction in frequency of cervico-vaginal HIV-1 RNA detection and plasma HIV-1 RNA load but no effect on viral load among those with detectable shedding [22]. Moreover, a trial of acyclovir (400 mg b.i.d.) delivered to the HIV positive partner in discordant couples (“Partners in Prevention”) found no effect on HIV transmission to their HIV negative partners, although there was some evidence that acyclovir reduced risk of HIV-1 disease progression to either CD4 count below 200 cells/µL, use of ART or death (hazard ratio(HR) = 0.84, 95%CI 0.71–0.98) [36].

Our results are consistent with previous reports of an association between HSV-2 shedding and HIV PVL, and suggest that clinical and subclinical herpetic reactivations might increase genital HIV shedding not only through a direct viral interaction at the genital level, but also through their systemic effect on plasma HIV viral load [37]. In our study, HIV plasma viral load was the strongest determinant of HIV genital shedding, and higher plasma viral loads were observed in women with herpetic ulcers and asymptomatic herpes than in uninfected women.

In this study we used cervicovaginal specimens as a marker of genital shedding, and did not find evidence of increased HIV shedding in the presence of genital lesions. However, HIV shedding may also occur directly from a herpetic lesion, and we cannot exclude the possibility that overall HIV infectivity is increased in the presence of lesions, particularly as 64% of herpetic lesions seen in this study were found on the vulva [38]. The quantification of both the HIV shedding and the HSV shedding were taken from the dilution of the CVS supernatant, and these are dependent on the amount of vaginal fluid in the vaginal lavage.

Genital specimens from women with ulcers of other confirmed aetiology (*T pallidum* or *H ducreyi*) were excluded from this analysis, as were specimens with traces of blood or semen, although we cannot rule out the possibility that some HIV or HSV detected in CVS originated from infected sexual partners. Some CVS were collected from women with non-ulcerative STI, and this may have led to underestimation of the association between herpetic lesions and HIV shedding, as cervicovaginal secretion of HIV-1 has been associated with the presence of non-ulcerative STI such as gonorrhoea and Chlamydia and found to decrease after effective treatment [39]–[41]). However, in this study the presence of other STI was not associated with increased HIV shedding (data not shown), and all symptomatic participants were given treatment for all STI free-of-charge throughout the study.

Increased genital HIV-1 viral load has been shown to be associated with immunosuppression in some studies [14], [41] but not others [42]. In our study, CD4 counts were not available, but most women had plasma HIV-1 RNA <30,000 copies/ml, and few specimens were collected at a time when lymph nodes or other clinical signs or symptoms of HIV-related disease were present. Furthermore, the HSV type was not determined in this study and some of the detected HSV may have been HSV-1 rather than HSV-2.

In conclusion, this study adds to the accumulating evidence that HSV-2 infection increases genital shedding of HIV, and may therefore increase the infectivity of HIV infected individuals to their sexual partners. Our data suggest that the increase in HIV shedding is related to the quantity of HSV shedding rather than to the presence of clinical lesions of herpes. These findings emphasise the importance of developing and evaluating effective interventions to control herpes in populations where HSV-2 may account for a substantial proportion of HIV transmission.
